# Assessment of creatinine concentration in whole blood spheroids using paper spray ionization–tandem mass spectrometry

**DOI:** 10.1038/s41598-022-18365-8

**Published:** 2022-08-22

**Authors:** Tung-Ting Sham, Abraham K. Badu-Tawiah, Stephen J. McWilliam, Simon Maher

**Affiliations:** 1 Department of Electrical Engineering and Electronics, University of Liverpool, Brownlow Hill, Liverpool, L69 3GJ UK; 2 Department of Chemistry and Biochemistry, Ohio State University, Columbus, OH USA; 3 Department of Women’s and Children’s Health, University of Liverpool, Institute in the Park, Alder Hey Children’s NHS Foundation Trust, Eaton Road, Liverpool, L12 2AP UK

**Keywords:** Nephrology, Kidney diseases, Analytical chemistry, Bioanalytical chemistry, Mass spectrometry, Mass spectrometry, Metabolomics

## Abstract

Accurate quantification of blood creatinine is important to estimate the glomerular filtration rate. Existing techniques using liquid chromatography tandem mass spectrometry (LC–MS/MS) have a high accuracy and eliminate most interferences encountered in routine enzymatic and Jaffé methods. However, they require laborious and time-consuming sample treatment and data acquisition. The aim of this study is to develop a fast and simple method to enable a direct analysis of whole blood creatinine with performance measures that are comparable to conventional LC–MS/MS. 5μL whole blood is formed as a three-dimensional spheroid on hydrophobic silanized paper substrates which then undergoes paper-spray ionization—tandem mass spectrometry (PSI–MS/MS). The method is validated using real human samples and compared with LC–MS/MS. PSI–MS/MS whole blood analysis exhibited a lower limit of quantification of 2.5 μg/mL, precision ≤ 6.3%, recovery in the range of 88–94% and excellent linearity (R^2^ > 0.99; 2.5—20 μg/mL) covering the normal range for creatinine levels. Creatinine levels were comparable to those measured by LC–MS/MS with small deviations of less than 0.3 μg/mL. This simple, fast and accurate microsampling technique for direct analysis of creatinine from whole blood shows promise for routine clinical screening and monitoring. This approach can be readily extended for other analytes of interest and, due to inherent advantages relating to cost, storability, speed, and simplicity, it can be especially advantageous for use in resource-limited settings.

## Introduction

Children with kidney diseases and undergoing kidney replacement therapy, require frequent blood tests to monitor their condition. The most common test performed is creatinine, used to estimate renal glomerular filtration rate^1^. Colorimetric assays using enzymatic and Jaffé methods are frequently used in clinical practice. However, these methods are prone to interference from hemolysis, lipemia, bilirubin, glucose, hemoglobin, and creatine^2,3^. The low-cost Jaffé method is still widely used. However, it is more susceptible to interferences and chromogens, requires critical time control, and creatinine levels might be under- or overestimated compared with other analytical methods^4,5^. Inaccuracy in the lower concentration range is of particular concern^6^ because of the potential to misclassify patients with respect to renal function.

There are a number of reported liquid chromatography-tandem mass spectrometry (LC–MS/MS) based methods that quantify creatinine in serum^7,8^, plasma^9^ and dried blood spots (DBS)^10^ with excellent specificity and sensitivity. LC–MS/MS eliminates interferences that react with the color reagent of colorimetric assays. However, this technique requires complicated and time-consuming sample pre-treatment: deproteination with organic solvents, centrifugation or filtration, followed by isolation of extracts, and lastly chromatographic separation and a costly LC column. Together, these drawbacks hinder urgent and immediate sample analysis.

DBS sampling has gained significant interest over the last few decades for bioanalysis, especially for newborn screening due to the use of smaller blood volumes (i.e., microsampling), stability of drugs and metabolites in dried blood, ease of specimen storage and transportation, and cost-effective sample collection^11,12^. Yet, manual handling of DBS samples is laborious. Whole blood that is freshly spotted onto filter paper generally needs a minimum of 2–3 h drying time in ambient conditions, followed by spot-punching for solvent extraction before LC–MS/MS. This sampling method suffers from the hematocrit effect^13^, paper chromatography effect (i.e. volcano effect)^14^, and risk of cross-contamination from a puncher^15^. Certainly, DBS sampling and associated handling methods require improvement.

Paper spray ionization–tandem mass spectrometry (PSI–MS/MS) for direct analysis of modified DBS enables rapid quantification of endogenous and xenobiotic compounds from whole blood samples^16–23^. The analyte solution is spotted onto a small triangular paper followed by the application of spray solvent and a high electric potential (> 3 kV), to instigate electrospray-like ionization^24–29^. Hence, this method integrates three main analytical procedures into one step: sample collection, analyte extraction, and analyte ionization, without the need for the time-consuming punching and extraction procedures of DBS. This method can allow on-site detection of biomarkers^18,30^ and therapeutic drugs^20,31^ from biological fluids, with excellent potential for point-of-care (POC) analyses to aid rapid clinical decision-making.

In this study, we present a refinement of classic PSI using a surface-modified hydrophobic paper substrate. This substrate enables the collection of whole blood which dries to form a three-dimensional spheroid. This promising technique has been shown to avoid volcanic effects and allow stable storage of labile constituents without the need for cold storage^19^. For example, exogenous cocaine in blood was shown to be labile when stored as a traditional DBS but stable when stored as a dried blood spheroid on a silanized paper substrate^19,32,33^. Analyte stability is attributed to a protective thin film that forms on the surface of the dried spheroid^33^. Whole blood storage and subsequent direct analysis show great promise, but basic clinical efficacy has not yet been demonstrated. For the first time, as a proof of concept, this technique has been significantly advanced and applied for microsample collection of whole blood from human adult volunteers to allow direct measurement of creatinine levels, which is cross-validated against an ultra-high performance liquid chromatography-tandem mass spectrometry (UPLC–MS/MS) assay providing a suitable foundation for future clinical studies.

## Materials and methods

### Chemicals and reagents

Creatinine (≥ 98%), reagent-grade formic acid (≥ 95%) and trichloro(3,3,3-trifluoropropyl)silane (97%) were purchased from Sigma–Aldrich (St. Louis, Mo, USA). Creatinine-D3 (purity ≥ 95%) was obtained from Toronto Research Chemicals (Canada). Methanol (99.8%, HPLC grade) and defibrinated horse whole blood (Oxoid Deutschland) were purchased from Fisher Scientific (Loughborough, UK). Water was purified using a Milli-Q Advantage A10 water purification system (Millipore*,* MA*,* USA) before use in this study. Chromatography paper (25 mm, Grade 1) was purchased from Whatman (Maidstone, UK).

### Preparation of stock, calibration solutions and spiked blood samples for standard addition

Standard stock solutions (1 mg/mL) of creatinine and internal standard, creatinine-D3, were prepared with 50% methanol in water and stored at − 20 °C. 400 μg/mL diluted creatinine standard solutions were further diluted to prepare working solutions at 0, 50, 100, 200, 300 and 400 μg/mL with water. For standard addition, 6 blood samples with spiked creatinine standard solutions at concentrations of 0, 2.5, 5 10 15 and 20 μg/mL were prepared by mixing 180 μL of whole blood with 10 μL creatinine-D3 (100 μg/mL) and 10 μL of creatinine working solutions. For the external calibration curve, a set of blank samples, mixed only with working solutions, were prepared using distilled water to replace the blood samples. All solutions were vortexed for 30 s and stored in a refrigerator at 2–8 °C before analysis.

### Hydrophobic paper preparation

Chromatography paper was cut into an isosceles triangular shape (surface area, about 40 mm^2^; base, 9 mm; height, 9 mm) with a digital template using a 40 W laser cutter at 20% speed and 10% cutting power (HPC Laser Ltd, UK). The triangular paper was washed with water, followed by methanol under sonication for 5 min and dried in a fume hood overnight. The hydrophobic paper was prepared by silanization method^18^. The dried triangular paper was silanized with 2 mL of trichloro(3,3,3-trifluoropropyl) silane in a vacuum desiccator for 1 h.

### Comparison of sample drying method

During the initial evaluation of the drying method, 5 μL of the same batch of whole blood, which was previously spiked with creatinine-D3 (5 μg/mL), was dried by three methods: in an oven at 37–40 °C, using a desiccator, and under ambient conditions. Drying times, average peak area ratios, and precision were evaluated in each case.

### PSI–MS/MS parameters

For paper spray, a copper clip was placed at the base of the triangular substrate to provide an electrical connection. The apex of the triangular paper was about 3 mm from the inlet of the mass spectrometer. The spray solution was 90% methanol in water with 0.1% formic acid which was automatically dispensed onto the top of the dried blood spheroid by a syringe pump during two distinct time frames at 0.01–0.07 min and 0.5–0.56 min, (about 30 μL volume per addition).

Mass spectrometry analysis was conducted with a Thermo Scientific Orbitrap Exploris 240 mass spectrometer (Thermo Fisher, Waltham, MA, USA). The mass-spectrometric parameters were set as follows: spray voltage, + 4 kV; ion transfer tube temperature, 400 °C without nebulizer gas supply. The selective reaction monitoring (SRM) transitions were: *m*/*z* 114.0662 → 44.049 (quantifier) and *m*/*z* 114.0662 → 86.071 (qualifier) for creatinine and *m*/*z* 117.0850 → 47.068 (quantifier) and *m*/*z* 117.0850 → 89.090 (qualifier) for creatinine-D3. The optimized absolute collision energy using higher energy collisional dissociation (HCD) for the four transitions noted were: 15, 10, 15 and 10 eV, respectively. The resolution was set at 60,000 per SRM transition. Internal mass calibration (EASY-IC) was applied. Scan time was 0.1 s per transition.

The voltage was applied after the first addition of spray solvent to induce an electrospray event for 18 s and stopped for 6 s. The voltage application cycle was repeated three more times to generate a total of four peaks in one chromatogram. The total run time was 2 min. Data processing was completed using the Xcalibur Quan Browser. Four peaks per sample were integrated and the mean peak area ratio of the quantifiers was used for quantification. All quantifications were performed with triplicate measurements unless specified otherwise.

### UPLC–MS/MS sample treatment and quantification

The accuracy of PSI–MS/MS for creatinine quantitation was compared against UPLC–MS/MS, which was modified from validated methods^34,35^. Deproteination of the same blood samples with methanol was conducted at the same time as PSI–MS sample treatment.

50 μL freshly collected and spiked whole blood sample with internal standard and calibration working solution was mixed with 150 μL cold methanol and vortexed for 30 s. The mixture stood at − 20 °C overnight for complete deproteination, followed by a further centrifuge at 18,700×*g* and − 4 °C for 20 min. 100 μL supernatant was transferred into an insert of amber HPLC vial and stored at − 20 °C prior to UPLC–MS/MS analysis. The dilution factor of the whole blood was 4.

3 µL aliquot was injected into a Waters ACQUITY UPLC system (Waters Corporation, Milford, MA). UPLC separation was performed on a Waters ACQUITY UPLC BEH C18 column (2.1 mm × 50 mm, 1.7 µm) with a BEH C18 guard column (2.1 mm × 5 mm, 1.7 µm). The mobile phase consisted of combinations of A (0.1% formic acid in water, v/v) and B (0.1% formic acid in acetonitrile, v/v) at a flow rate of 0.3 mL/min with an elution gradient as follows: 0–1 min, 5% B; 1 min, 95% B; 3 min, 95% B. A 2.5-min post-run time was set to fully equilibrate the column. Column and sample chamber temperatures were 40 °C and 8 °C respectively.

Mass spectrometry analysis was conducted by a Waters Xevo Triple mass spectrometer (Waters, Milford, USA) with electrospray ionization (ESI) in positive mode. Nebulization and cone gases were both nitrogen and, set at 800 L/h and 150 L/h, respectively. The source temperature was kept at 450 ℃. The source capillary voltage was 3.7 kV. Argon was applied as collision gas. The multiple reaction monitoring (MRM) transitions monitored were: *m*/*z* 114.01 → 43.97 as a quantifier and *m*/*z* 114.01 → 86.05 as a qualifier for creatinine and *m*/*z* 117.04 → 47.03 as quantifier and *m*/*z* 117.04 → 89.07 as qualifier for creatinine-D3. The optimized parameters for the four MRM transitions were: cone voltage, 26, 26, 20 and 20 V; collision energy: 13, 11, 13 and 11 eV, respectively. The dwell time was 0.147 s per transition. Peaks were integrated and the peak area ratio of the quantifiers of creatinine and creatinine-D3 were used for quantification.

### Method validation and statistical analysis

External calibration curves and standard addition curves were constructed by plotting peak area ratios of creatinine to that of creatinine-D3 versus the theoretical concentration. Calibration curves were acceptable if the correlation coefficient was > 0.99 and accuracies (bias, %) were within 20% of the theoretical value for the lower limit of quantification (LLOQ) and < 15% of the theoretical values for the remaining standards, based on Guidance for Industry: Bioanalytical Method Evaluation^36^.

Due to the lack of creatinine-free blood samples, limits of detection (LOD) and LLOQ were obtained from the calibration curve of peak area ratios of creatinine-D3 to that of endogenous creatinine with regard to theoretical concentrations (0.5–20 μg/mL) from the same batch of horse blood. The LOD was calculated as: (3.3 × SD of the y-intercept of calibration curve) / the slope of the trend line^37^. The LLOQ is the lowest concentration that can be measured with acceptable accuracy (80–120%) and precision (± 20%), and with 5 times the response compared to a blank response^36^. Precision relates to the coefficient variation of concentrations determined from multiple replicates taken from a single horse whole blood sample. Recovery was tested by spiking the same batch of horse whole blood with creatinine at low, middle, and high concentrations as well as creatinine-D3 in triplicate and analyzed as noted above, in comparison with the amount obtained by the external calibration curve. Qualifier/quantifier ion ratios and matrix effects were also studied and the corresponding method details are given in the Supplementary materials, Sects. 1.1 and 1.2.

Statistical analyses were performed using IBM SPSS Statistics version 25 (Chicago, IL, USA). Statistical differences were evaluated by Student’s *t*-test and one-way ANOVA with *Tukey HSD* post-hoc test at a univariate level. A *p*-value < 0.05 was considered to be statistically significant.

### Cross-validation using real human samples

Three whole blood specimens without anti-coagulants were donated by three healthy volunteer adults (NHS staff) using venipuncture, following written, informed consent. Ethical approval was granted by the University of Liverpool Central University Research Ethics Committee C (REC: 6386) and all procedures were in accordance with the Helsinki Declaration. Aliquoted whole blood was transferred into lithium heparin tubes, and then centrifuged at 5000×*g* for 10 min at 4 °C to isolate plasma. 180 μL freshly collected whole blood and plasma specimens were spiked with 10 μL creatinine-D3 and 10 μL of creatinine working solutions (same as “Preparation of stock, calibration solutions and spiked blood samples for standard addition”). 5 μL and 50 μL of each aliquot were tested simultaneously with PSI–MS/MS and UPLC–MS/MS, respectively, for creatinine quantification.

### Scanning electron microscope (SEM) imaging

The surface characteristics of dried blood spheroids under different drying conditions were studied with a SEM. Dried whole blood spheroids deposited on paper substrates were coated with a thin layer of gold (10 nm). SEM images were acquired using a JEOL JSM 7001F SEM system under vacuum with an accelerating voltage of 10 kV.

## Results

### Substrate optimization

**Figure 1:**
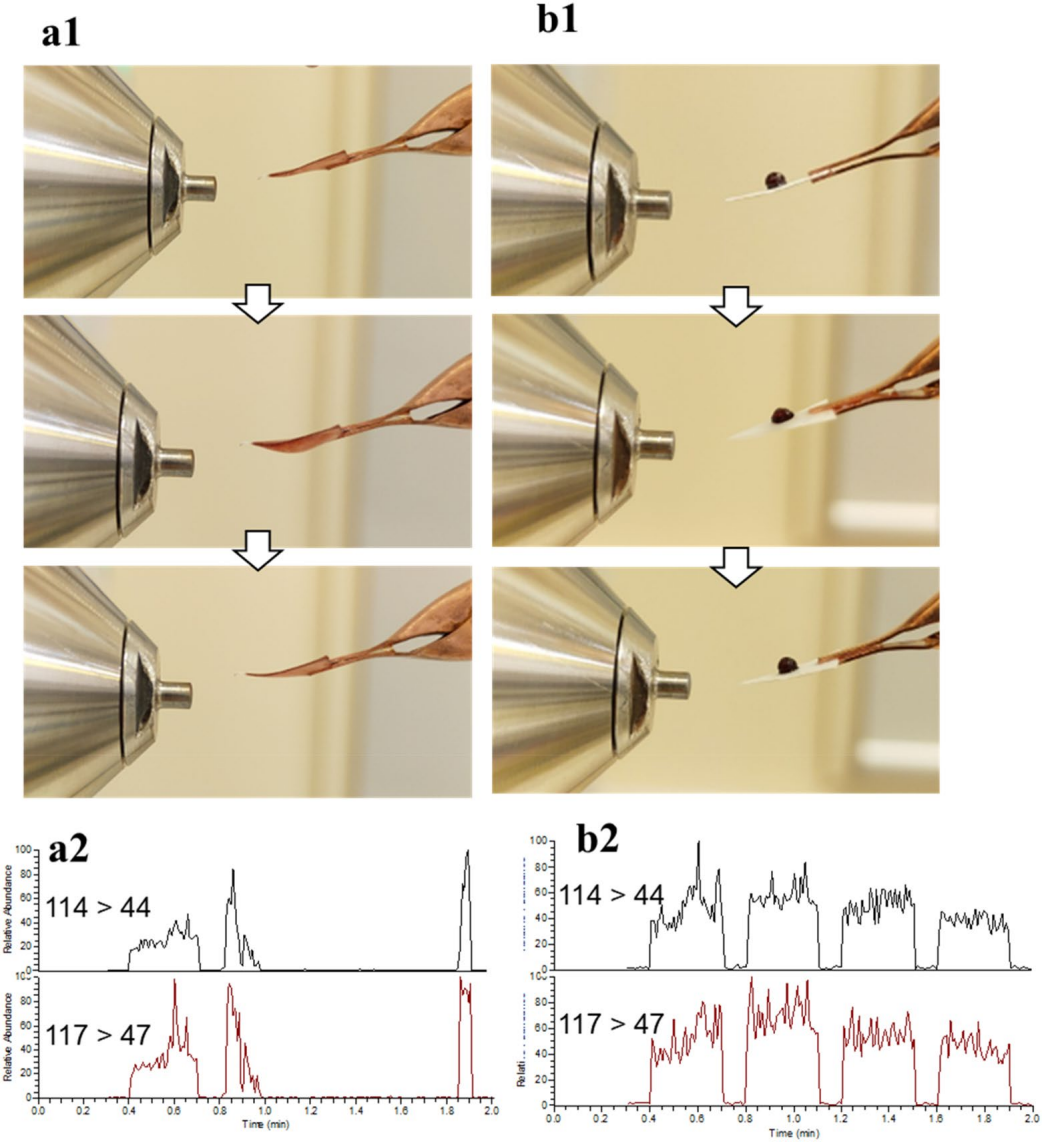
(1) Comparing change of paper morphology after addition of spray solvent and electro-spraying during PSI–MS/MS analysis and (2) SRM chromatograms of creatinine and creatinine-D3 quantifiers between (**a**) normal unsilanized (hydrophilic) paper and (**b**) silanized (hydrophobic) paper.

Trichloro(3,3,3-trifluoropropyl) silanized hydrophobic paper allows blood to be collected as three-dimensional (3D) spheroids rather than two-dimensional (2D) dried spots^19^. The water contact angle of the hydrophobic paper was evaluated and found to be 115.2 ± 3.5° (*n* = 10) (Supplementary Fig. S1b1). The hydrophobic paper was found to have improved spray stability (Fig. 1). Whilst a key aim of this study is to evaluate clinical efficacy of hydrophobic PS-MS with real human samples for the collection and storage of whole blood as a 3D spheroid rather than a 2D spot, some comparison is made with traditional dried blood spot PS-MS for added context. When testing traditional (i.e., unsilanized) paper spray, there was a tendency for the tip of the paper to curl upwards during electrospray (Fig. 1a1). However, this phenomenon was not observed with hydrophobic paper (Fig. 1b1). This curling effect had an impact on the spray process and signal stability (Fig. 1a2,b2). The curling of un-silanized paper might be due to the moisture absorbance from the spray solvent (90% methanol with 10% water) and then expansion of the cellulose fibers from the untreated (hydrophilic) paper, resulting in curling at the tip of the paper. We surmise that silanization of the hydroxyl group of cellulose fibers results in moisture diffusing uniformly on the surface of the hydrophobic paper, leading to stable sprays. This is further supported by our SEM images of the silanized paper which showed a reduction in the number of pores and fibers (Supplementary Fig. S1b2–4).

### Optimization of drying methods

**Figure 2:**
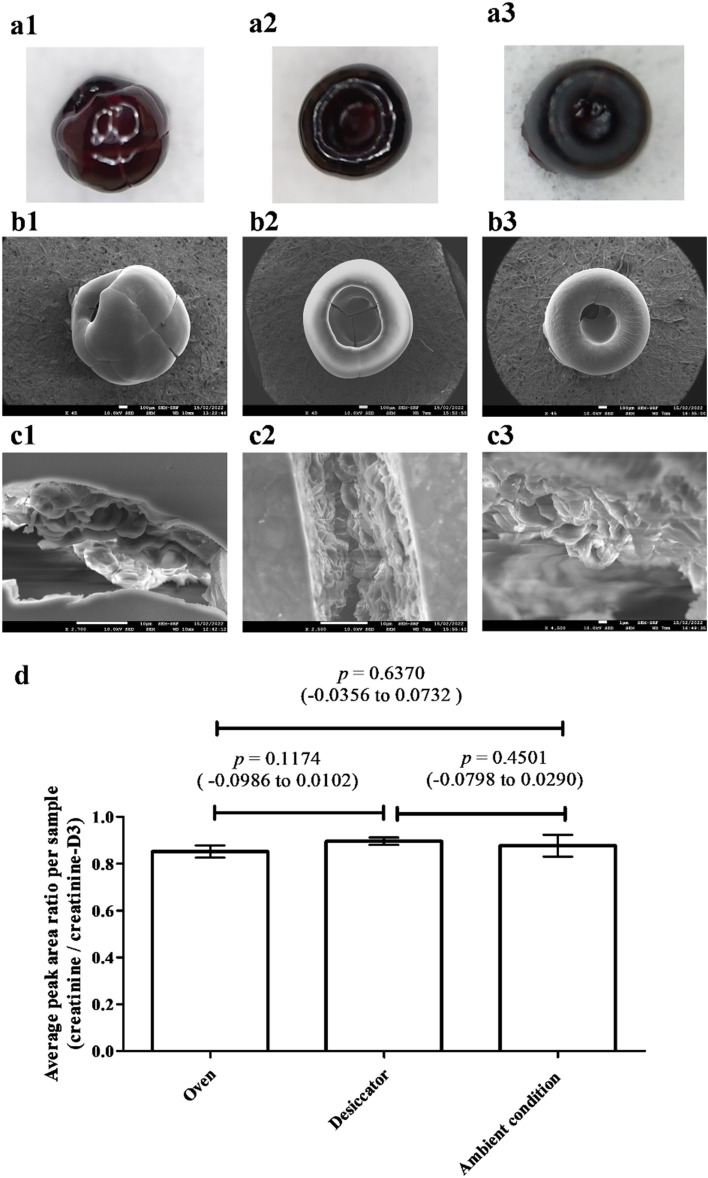
(**a**) Photographs of real human whole blood spheroids (× 45) for different drying conditions: (1) in an oven for 12 min, (2) desiccator for at least 1 h, and, (3) ambient condition for at least 1.5 h. (**b**) SEM top views and (**c**) interior view of the cracks. (**d**) Peak area ratios of spiked creatinine to creatinine-D3 from whole blood dried under the different conditions. *p* value: one-way ANOVA with post-hoc *Tukey HSD* test (95% confidence interval).

From the SEM imaging (Fig. 2a,b), oven-dried spheroids showed a more spherical shape in the central region without obvious buckling. Desiccator- and air-dried spheroids had obvious buckling from their top views. Air-dried spheroids also showed more ridges formed on the surface, possibly due to slow evaporation, whereas more small cracks were evident at the center of the desiccator-dried spheroids^38^.

Despite the visible differences on the surface of the dried spheroids, the different drying conditions had no impact on the stability of creatinine. The peak area ratios obtained from samples dried in the oven or under ambient conditions had no statistically significant difference compared to the desiccator-drying method (*p* > 0.05) with CV = 3.1%, 5.3% and 1.7%, respectively (Fig. 2d). The duration taken for the complete drying of the blood spheroids in the oven was just 12 min while those in the desiccator or left under ambient conditions required at least 1 and 1.5 h, respectively. To shorten the sample preparation time, the oven-drying method at 40 °C for 12 min was selected.

### Method validation

#### Interference

The purity of creatinine and creatinine-D3 were confirmed by in-house UPLC–MS. They were free from contaminants. Neither of them was detected in the blood collection tube, paper substrate or extraction solvent. The qualifier/quantifier ion ratios of creatinine and creatinine-D3 measured in human blood spheroid samples used for the standard addition were 6.8% ± 0.7% and 3.9% ± 0.4%, respectively (Supplementary Table S1). This is within permitted tolerances (± 20% of the average of neat calibration solutions^39^), indicating the absence of interferences in transition ions from the blood samples. Representative PSI–MS/MS chromatograms and spectra of the blank water, standard solution and a human blood sample are shown in Supplementary Figs. S4 and S5.

#### Limits of detection and quantification

Given the endogenous nature of creatinine, in order to determine its LOD, we used an internal standard, creatinine-D3, an isotopologue of creatinine, that shares the same chemical structure and ionization efficiency, as a substitute for the analyte. The endogenous creatinine in the blood was used for the normalization of creatinine-D3. The PSI–MS/MS assay of creatinine-D3 spiked in horse whole blood spheroids showed a linear range of 0, 2.5–20 μg/mL with R^2^ = 0.9902 (Supplementary Fig. S2), with LLOQ bias of 2.5 μg/mL at 7.0–14.4%, and bias of other concentrations at − 1.1–8.4%. The precision of peak area ratios obtained from triplicates of all concentrations was 1.3–3.2%. Based on this calibration curve, the LOD and LLOQ were estimated to be 0.919 μg/mL and 2.5 μg/mL, respectively.

#### Linearity of the response

Additionally, the linearity of spiking creatinine in the blood (standard addition) and pure water (external calibration) was evaluated by analyzing samples prepared within the concentration range of 0.5–20 μg/mL (Supplementary Fig. S3). The standard addition of creatinine spiked whole blood between 2.5 and 20 μg/mL showed linearity of R^2^ = 0.9967 with bias at 9–10%. The external calibration curve without blood matrix between 0.5 μg/mL and 20 μg/mL showed linearity of R^2^ = 0.9951 with bias of the LLOQ (0.5 μg/mL) at 7.9–16.3% and bias for other concentrations at 1.3–7.4%. Our linearity range covers normal adult serum creatinine levels^40^ [0.6–1.2 mg/dL (6–12 μg/mL) for men and 0.5–1.1 mg/dL (5–11 μg/mL) for women]**.**

#### Precision and recovery

The intra-day and inter-day precision peak area ratios of creatinine to creatinine-D3 at zero, low, middle and high spiked concentrations were evaluated as, CV = 1.9–4.3% (*n* = 5 per day) and 3.7–6.3% (3 days), respectively, as shown in Table 1. This is well within the acceptable range (CV ± 20%). The recovery from spiking three concentrations of creatinine into horse whole blood was 88–94% (CV ≤ 8.6%) within the acceptable range of 80–120%, as summarized in Table 1.

**Table 1:** Precision and recovery of horse whole blood spheroids analysis by PS-MS/MS.

	Spiked creatinine concentrations			
	0 μg/mL	2.5 μg/mL	5 μg/mL	10 μg/mL
**Intra-day precision (n = 5)**				
Average peak area ratio	0.649	0.850	1.024	1.423
SD	0.023	0.016	0.044	0.034
CV, %	3.6	1.9	4.3	2.4
**Interday precision (n = 4 / day, 3 days)**				
Average peak area ratio	0.657	0.847	1.034	1.383
SD	0.034	0.037	0.038	0.087
CV, %	5.2	4.3	3.7	6.3
**Recovery (n = 3)**				
Measured total spiked and blood amount (μg/mL)	8.03	10.22	12.62	17.47
SD	0.21	0.11	0.05	0.86
Measured spiked amount (μg/mL)		2.19	4.58	9.44
SD		0.14	0.05	0.86
Recovery, %		88	92	94
CV, %		4.5	0.9	8.6

#### Matrix effect

A comparison of the internal standard signal response in the dried blood spheroid against that in blank water showed an average ion suppression of 87 ± 1% (Supplementary Table S2). The matrix effect on creatinine signal response could be minimized by spiking its internal standard in all blood samples before all PSI–MS/MS runs, followed by the use of the peak area ratio of creatinine to the internal standard for quantification.

### Cross-validation using real sample analysis

To verify the proposed blood sampling method and analysis for clinical use, the described protocol was applied to the analysis of whole blood samples from healthy volunteers (NHS staff).

**Figure 3:**
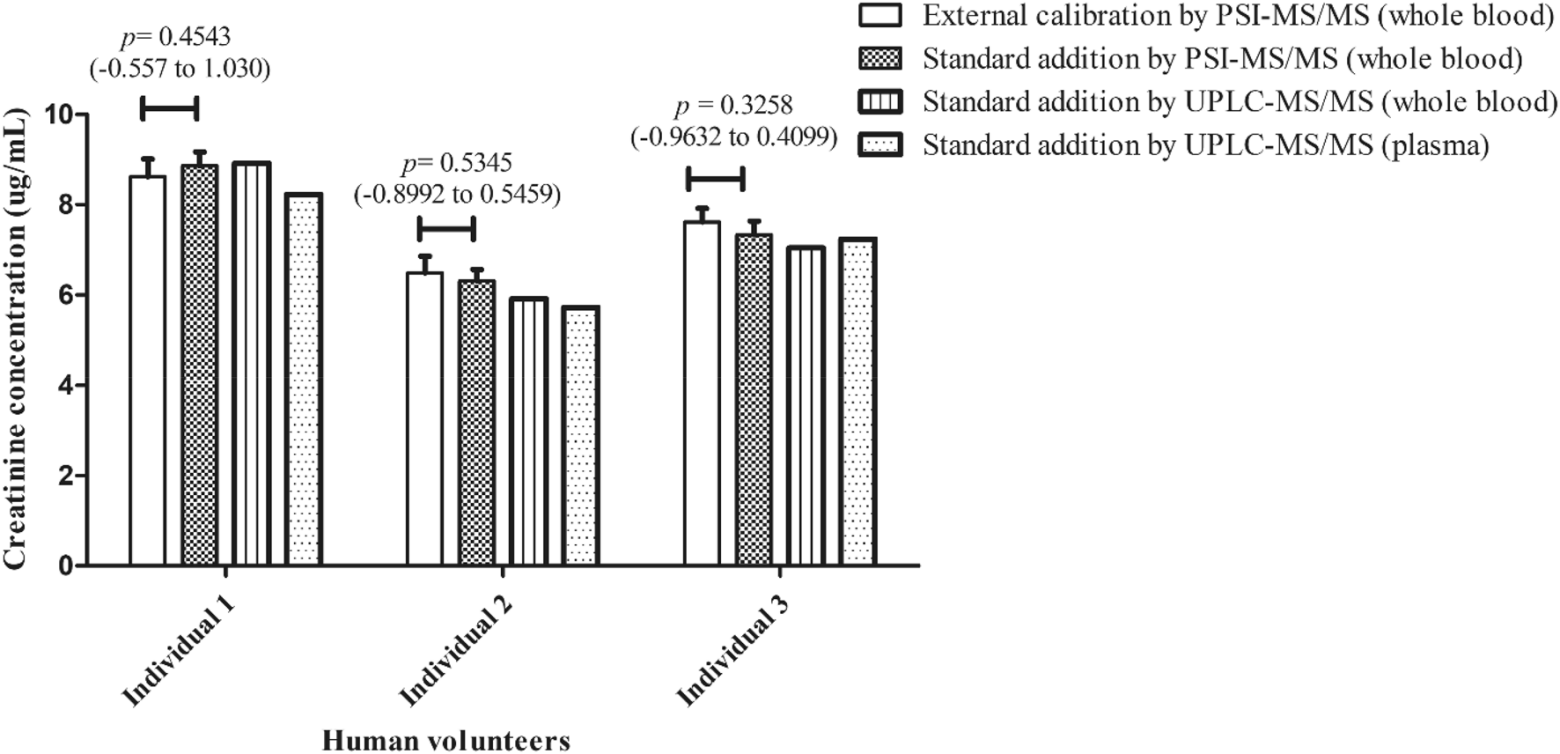
Comparison of creatinine concentrations in real human blood samples between different calibration and mass spectrometry methods. *p* value: Student’s t-test (95% confidence interval).

**Figure 4:**
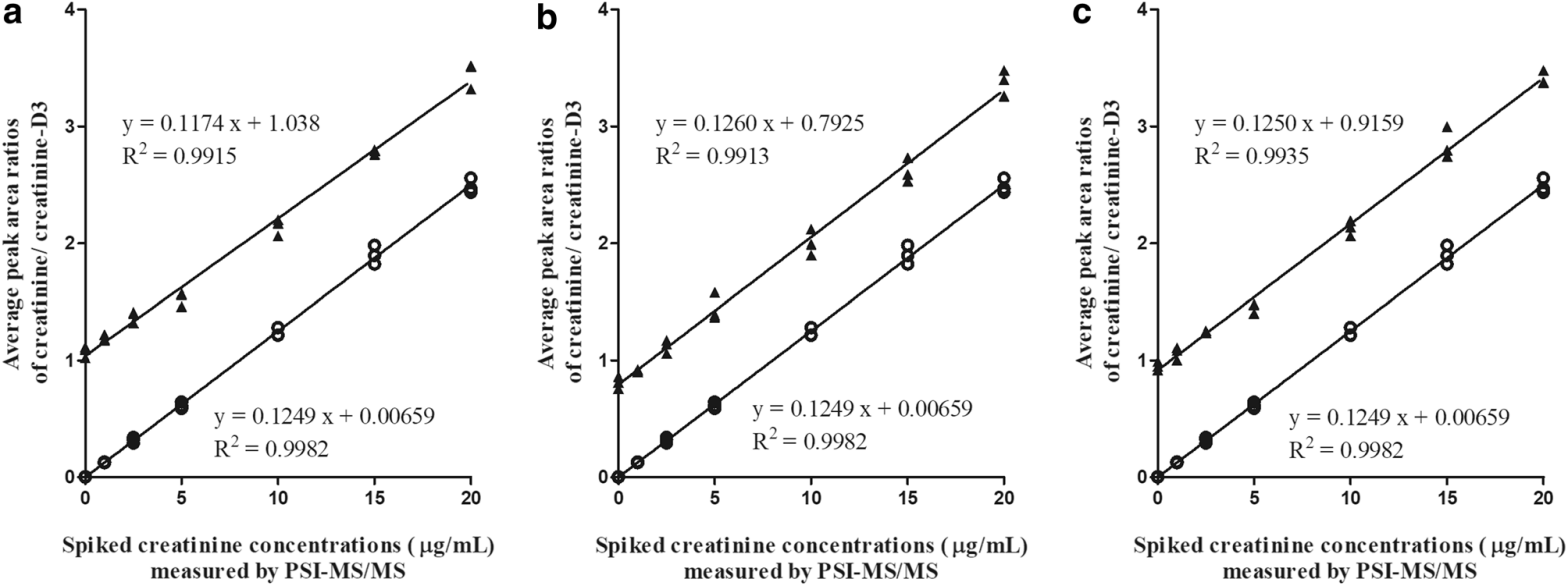
Comparison of calibration curves by PSI–MS/MS using pure standards and spiked blood samples of three volunteers (**a** individual 1; **b** individual 2; **c** individual 3). Curves with circle symbols: pure standards (external calibration); curves with triangle symbols: spiked blood samples (standard addition).

To assess the effect of the dried blood matrix on the PSI–MS/MS assay of creatinine, two creatinine concentrations from the same blood sample were calculated, using the calibration curve equations obtained with neat calibration solutions (external calibration) and standard-spiked blood mixtures (standard addition), respectively, and compared. As summarized in Fig. 3 and Supplementary Table S3, the two methods showed comparable results (differences: − 0.07 ± 0.28 μg/mL). Their calibration curves showed similar slopes and were parallel to each other (Fig. 4). In addition, the silanized paper showed a similar quantification result as the unsilanized counterpart based on the same external calibration curve (*p* > 0.05) (Supplemental Fig. S6).

To evaluate the quantification of creatinine using the PSI-MS/MS method (directly from human whole blood), the results were compared against a state-to-the-art UPLC–MS/MS method, which was simultaneously applied to two types of blood samples, plasma and whole blood. Representative UPLC–MS/MS chromatograms are shown in Supplementary Fig. S7. The results obtained by PSI–MS/MS, using both external calibration and standard addition, had small deviations from those detected by UPLC–MS/MS; deviation = 0.28 ± 0.50 μg/mL and 0.21 ± 0.24 μg/mL compared with whole blood samples; 0.51 ± 0.22 μg/mL and 0.60 ± 0.03 μg/mL compared with plasma blood samples (Supplementary Table S3). The linearity obtained for PSI–MS/MS, (R^2^ = 0.9951–0.9967), is comparable with that of UPLC–MS/MS, (R^2^ = 0.9927–0.9977), as shown in Supplementary Fig. S8.

## Discussion

Our study assessed the potential of PSI–MS/MS for direct analysis and quantification of creatinine from whole blood spheroids, in comparison with conventional UPLC–MS/MS. Considering the simplicity and speed of the procedure, PSI–MS/MS analysis exhibits excellent intra-day and inter-day precision and recovery.

Assessment of three drying methods, to allow the blood spheroid to form, showed the merits of a mild and fast oven-drying process which reduces the time required for dehydration 4-6x (compared to ambient drying procedures). It also did not cause thermal or oxidative degradation of creatinine in the spheroid. Although the oven-dried spheroid exhibits a different exterior appearance from the other two methods, this did not affect analytical performance. Interestingly, red blood cells were observed on the interior surface of spheroid cracks in all three drying methods (Fig. 2c), indicating that the entire red blood cells did not lyse during the three drying processes.

In this study, method comparison between PSI–MS/MS and UPLC–MS/MS analyses showed good consistency using human samples. Whilst the sample size is very small, this promising proof of concept result provides a basis for more extensive studies. The small differences between PSI–MS/MS analysis of dried blood spheroids and UPLC–MS/MS analysis of plasma derived from the same samples indicate that the presence of blood cells is not detrimental to creatinine quantification. The creatinine quantification performance of both external calibration and standard addition methods were very close to each other (deviation < 0.6 μg/mL, Fig. 3). By examining the chromatograms for the blank solution or spiked blood spheroid, it is clear that the ion signals relating to the internal standard changed similarly to those of creatinine (Supplemental Fig. S1). The internal standard successfully normalized the signal of creatinine with a low CV (22–48% reduced to 2.8–7%). This similarity indicates that when using a standard addition method with spiked whole blood, the physical barriers on the spheroid surface (dried red blood cells) and indeed the wider whole blood matrix, did not affect the extraction or the signal normalization of the internal standard for spiked and un-spiked creatinine. The recovery was high (88–94%) in different spiked concentrations.

This study has set out to assess the potential of this approach for routine clinical analysis, in comparison to an accepted state-of-the-art method. Whilst this approach shows significant promise, there are some limitations that should be commented upon and addressed in future work. The real sample analysis was limited to only three healthy adults (NHS staff volunteers). Whilst sufficient for an initial early-stage proof of concept, a large population covering a wide range of ages and BMI should be included in any similar or follow-up studies to obtain a broader concentration range. The LOD and LOQ were calculated based on the internal standard instead of creatinine, due to the unavailability of a creatinine-free (i.e., blank) whole blood matrix; the accuracy of the calibration curve was estimated and found to be within an acceptable range (< 15%). In addition, for the particular PSI–MS/MS method developed herein, the average peak area ratios from four spray signals of the same paper were used instead of one spray. Whilst this makes little difference to the timescale of the analysis (an additional 1.3 min), it was deemed necessary because the initial extraction following the first dispensing of the spray solvent was found to be suboptimal due to the presence of dried platelets, fibrin and red blood cells at the surface of dried human blood spheroid^33,43^.

For this method to be used for routine clinical analysis, other practicalities should be considered. A preliminary cost and time analysis comparing PSI–MS/MS and UPLC–MS/MS (as well as Jaffé and enzymatic methods) has been summarized in Supplementary Table S4. In terms of the average cost and time expended per sample analysis, it is estimated that PSI–MS/MS is the most cost- and time-effective (approximately ~ 10 × more than UPLC–MS/MS). It is well-known that UPLC–MS/MS assays can require large amounts of time for sample preparation and pre-conditioning, and this, in part, is responsible for the excellent analytical performance obtained. In this particular case, the UPLC–MS/MS method used requires >  ~ 80 min to remove protein from each blood sample. The method also, generally, requires a skilled practitioner as well as time for optimization of the LC gradient and pre-conditioning of the LC–MS system. Whereas, the simple and rapid sample preparation for the PSI–MS/MS method developed herein does not require significant labor nor extensive training to conduct. Conceivably, with a portable mass spectrometer, it would also be possible to offer this method in a general clinical setting without any expert assistance required. Another consideration for clinical practice relates to automation. In this study, good reproducibility, sufficient for routine clinical analysis, has been demonstrated even with a manual approach. This bodes well for the future as one would fully expect reproducibility to improve even further when automated due to increased positional accuracy and elimination of human errors, besides other additional benefits such as shorter sample-handling time and increased throughput.

Future work regarding the PSI–MS/MS method developed herein will also consider the volume of whole blood collected and inclusion of the internal standard. In this study, from the intravenous blood collection, we mixed 190 μL of whole blood with 10 uL internal standard, before dispensing 5 μL onto a paper substrate. This ensures a homogenous distribution of the internal standard throughout the blood sample without significant dilution of metabolites. Other options for inclusion of an internal standard exist, such as applying it directly onto the paper before or after dispensing blood onto the paper, or it can be included during the analysis (i.e., during the transient phase of spheroid wetting). Such considerations will be important if point-of-care (POC) analysis is to be conducted on-site (e.g., finger-prick microsample analysis with a portable mass spectrometer).

## Conclusion

We have developed a new method for PSI–MS/MS-based quantification of creatinine directly from an oven-dried 5μL whole blood spheroid. The microsampling and subsequent direct analysis allows for fast and effective quantification. This proof-of-concept clinical study has demonstrated that the procedure is practical, simple, fast and effective relative to the current state of the art, covering the expected normal human serum concentration range. Moreover, it is estimated to be ~ ten times more cost- and time- effective, with minimal environmental impact. Further studies involving larger sample sets and more diverse populations are required to verify its application in clinical use, especially for pediatrics. Due to the nature of the blood spheroid that forms and the inherent protection it affords (e.g., from oxidative degradation) negating the requirement for cold storage, this method also offers potential for ‘greener’ storage/analysis, especially in resource-limited healthcare settings.

## Supplementary Information

Supplementary Information.

## Data Availability

The data that support the findings of this study are available from the corresponding author upon reasonable request.

## References

[1] Mian AN, Schwartz GJ (2017). Measurement and estimation of glomerular filtration rate in children. Adv. Chronic. Kidney Dis..

[2] Bernstone L, Jayanti A, Keevil B (2019). A simplified, rapid LC-MS/MS assay for serum and salivary creatinine. Clin. Mass Spectrom..

[3] Peake M, Whiting M (2006). Measurement of serum creatinine–current status and future goals. Clin. Biochem. Rev..

[4] Liu WS (2012). Serum creatinine determined by Jaffe, enzymatic method, and isotope dilution-liquid chromatography-mass spectrometry in patients under hemodialysis. J. Clin. Lab. Anal..

[5] Walsh DA, Dempsey E (2002). Comparison of electrochemical, electrophoretic and spectrophotometric methods for creatinine determination in biological fluids. Anal. Chim. Acta.

[6] Hoste L, Deiteren K, Pottel H, Callewaert N, Martens F (2015). Routine serum creatinine measurements: How well do we perform?. BMC Nephrol..

[7] Ou M (2015). LC-MS/MS method for serum creatinine: Comparison with enzymatic method and Jaffe method. PLoS ONE.

[8] Sukhang M, Junkuy A, Buckley N, Mohamed F, Wunnapuk K (2020). An LC-MS/MS method for creatine and creatinine analysis in paraquat-intoxicated patients. J. Environ. Sci. Health B.

[9] Zhao Y (2016). A validated LC–MS/MS method for the quantitative measurement of creatinine as an endogenous biomarker in human plasma. Bioanalysis.

[10] Nakano M (2017). Development of tandem mass spectrometry-based creatinine measurement using dried blood spot for newborn mass screening. Pediatr. Res..

[11] Zakaria R, Allen KJ, Koplin JJ, Roche PJ, Greaves RF (2016). Advantages and challenges of dried blood spot analysis by mass spectrometry across the total testing process. EJIFCC.

[12] Lim MD (2018). Dried blood spots for global health diagnostics and surveillance: Opportunities and challenges. Am. J. Trop. Med. Hyg..

[13] Daousani C, Karalis V, Malenović A, Dotsikas Y (2019). Hematocrit effect on dried blood spots in adults: A computational study and theoretical considerations. Scand. J. Clin. Lab. Invest..

[14] Wagner M, Tonoli D, Varesio E, Hopfgartner G (2016). The use of mass spectrometry to analyze dried blood spots. Mass Spectrom. Rev..

[15] Murphy SC, Daza G, Chang M, Coombs R (2012). Laser cutting eliminates nucleic acid cross-contamination in dried-blood-spot processing. J. Clin. Microbiol..

[16] Yannell KE, Kesely KR, Chien HD, Kissinger CB, Cooks RG (2017). Comparison of paper spray mass spectrometry analysis of dried blood spots from devices used for in-field collection of clinical samples. Anal. Bioanal. Chem..

[17] Espy RD (2014). Paper spray and extraction spray mass spectrometry for the direct and simultaneous quantification of eight drugs of abuse in whole blood. Anal. Chem..

[18] Damon DE (2016). Direct biofluid analysis using hydrophobic paper spray mass spectrometry. Anal. Chem..

[19] Damon DE (2018). Dried blood spheroids for dry-state room temperature stabilization of microliter blood samples. Anal. Chem..

[20] Espy RD, Manicke NE, Ouyang Z, Cooks RG (2012). Rapid analysis of whole blood by paper spray mass spectrometry for point-of-care therapeutic drug monitoring. Analyst.

[21] Smith BL (2022). Ambient ion focusing for paper spray ionisation. Int. J. Mass Spectrom..

[22] Maher S, Jjunju FPM, Taylor S (2015). Colloquium: 100 years of mass spectrometry: Perspectives and future trends. Rev. Mod. Phys..

[23] Damon DE (2016). 2D wax-printed paper substrates with extended solvent supply capabilities allow enhanced ion signal in paper spray ionization. Analyst.

[24] Jjunju FPM (2020). Analysis of non-conjugated steroids in water using paper spray mass spectrometry. Sci. Rep..

[25] Sarih NM (2020). Accelerated nucleophilic substitution reactions of dansyl chloride with aniline under ambient conditions via dual-tip reactive paper spray. Sci. Rep..

[26] Suraritdechachai S (2019). Rapid detection of the antibiotic sulfamethazine in pig body fluids by paper spray mass spectrometry. J. Agric. Food Chem..

[27] Maher S (2016). Direct analysis and quantification of metaldehyde in water using reactive paper spray mass spectrometry. Sci. Rep..

[28] Jjunju FPM (2016). Screening and quantification of aliphatic primary alkyl corrosion inhibitor amines in water samples by paper spray mass spectrometry. Anal. Chem..

[29] Damon DE (2019). Determining surface energy of porous substrates by spray ionization. Langmuir.

[30] Yang Q (2012). Direct and quantitative analysis of underivatized acylcarnitines in serum and whole blood using paper spray mass spectrometry. Anal. Bioanal. Chem..

[31] Shi R-Z, El Gierari ETM, Manicke NE, Faix JD (2015). Rapid measurement of tacrolimus in whole blood by paper spray-tandem mass spectrometry (PS-MS/MS). Clin. Chim. Acta.

[32] Frey BS, Heiss DR, Badu-Tawiah AK (2022). Embossed paper platform for whole blood collection, room temperature storage, and direct analysis by pinhole paper spray mass spectrometry. Anal. Chem..

[33] Frey BS (2021). Protective mechanism of dried blood spheroids: Stabilization of labile analytes in whole blood, plasma, and serum. Analyst.

[34] Hou H (2012). LC-MS-MS measurements of urinary creatinine and the application of creatinine normalization technique on cotinine in smokers' 24 hour urine. J. Anal. Methods Chem..

[35] Zahoor N, Danilenko U, Vesper HW (2019). A fully automated high-throughput liquid chromatography tandem mass spectrometry method for measuring creatinine in urine. Clin Mass Spectrom.

[36] U.S. Food and Drug Administration, Center for Drug Evaluation and Research (CDER) & Center for Veterinary Medicine (CVM). Guidance for industry: Bioanalytical method validation. (2018).

[37] ICH harmonized tripartite. Guideline. Validation of analytical procedures: text and methodology Q2(R1). (2005).

[38] Iqbal R, Shen AQ, Sen AK (2020). Understanding of the role of dilution on evaporative deposition patterns of blood droplets over hydrophilic and hydrophobic substrates. J. Colloid Interface Sci..

[39] Drugs, U. N. O. o., Laboratory, C. & Section, S. *Guidance for the validation of analytical methodology and calibration of equipment used for testing of illicit drugs in seized materials and biological specimens: a commitment to quality and continuous improvement*. (United Nations Publications, 2009).

[40] Hosten, A. O. *Chapter 193 BUN and Creatinine*. 3rd edn, (Butterworths, 1990). 21250147

[41] Levey AS (2007). Expressing the modification of diet in renal disease study equation for estimating glomerular filtration rate with standardized serum creatinine values. Clin. Chem..

[42] Jelani, I. B. J., Abbas, H. K., Yale, B. M., Abacha, F. Z., Abdullahi, H. L. Comparison between jaffe and enzymatic creatinine assays in renal dysfunction subjects. *Saudi J. Med. Pharmaceut. Sci.* **7**, 267–269. 10.36348/sjmps.2021.v07i06.007 (2021).

[43] Tutwiler V (2016). Kinetics and mechanics of clot contraction are governed by the molecular and cellular composition of the blood. Blood.

