# Supplementary material for: Assessment of creatinine concentration in whole blood spheroids using paper spray ionization–tandem mass spectrometry
Source: Sci Rep. 2022 Aug 22;12:14308. doi: 10.1038/s41598-022-18365-8 (PMC9395369; doi:10.1038/s41598-022-18365-8)

Supplemental Information

Assessment of Creatinine Concentration in Whole Blood Spheroids using Paper Spray Ionization - Tandem Mass Spectrometry

Tung-Ting Sham [1], Abraham K. Badu-Tawiah [2], Stephen J McWilliam *[3], and Simon Maher *[1]

[1] Department of Electrical Engineering and Electronics, University of Liverpool, Liverpool, UK.
[2] Department of Chemistry and Biochemistry, Ohio State University, Ohio, USA.
[3] Department of Women's and Children's Health, University of Liverpool, Liverpool, UK.

*Address correspondence to:
S. Maher at Department of Electrical Engineering and Electronics, University of Liverpool, Brownlow Hill, Liverpool, L69 3GJ, UK. E-mail: s.maher@liverpool.ac.uk
S. McWilliam at Department of Women's and Children's Health, University of Liverpool, William Henry Duncan Building, 6 West Derby Street, Liverpool, L7 8TX, UK. E-mail: stevemcw@liverpool.ac.uk

Keywords: dried blood spots, paper spray, ambient ionization, microsample, creatinine, whole blood analysis, mass spectrometry, hydrophobic paper.

Supplementary information

1. Methods

1.1. Qualifier/quantifier ion ratios

Deviation in the qualifier/quantifier ion ratios often indicates the presence of interferences in the samples. The qualifier/quantifier ion ratios for the peak area of creatinine and that of creatinine-D3 were measured in human blood spheroid samples used for standard addition and compared with the average ratios of neat calibration solutions.

1.2. Matrix effect in the presence of dried blood spheroid matrix

To evaluate any signal suppression or enhancement caused by co-spraying dried blood spheroid matrix, we compared the signal intensities of the same standard in the presence and absence of dried horse whole blood spheroid. Since there is no creatinine-free matrix available, an internal standard, creatinine-D3 was used for the evaluation. In order to remove the effect of the extraction barrier from the dried blood spheroid, 5µL internal standard in water at concentrations of 2.5, 5, 10 and 20 µg/mL were added behind the dried blood spheroid, respectively, instead of mixing together to ensure it moved together with the blood matrix without extraction barrier. They were also added on a blank hydrophobic paper at a similar location as the blood matrix. After oven-drying, they were subjected to PSI-MS/MS analysis and their signal responses were compared. The test was performed in triplicate.

Supplemental Table S1 Ratios of qualifier/ quantifier ions from neat calibration solutions and a human whole blood sample spiked with creatinine and creatinine-D3 used for standard addition, expressed in percentages.

	Ratios of qualifier/ quantifier		
**Neat calibration solutions**	**Average peak area ratios**	**SD**	**CV**
**Creatinine (0, 2.5–20 µg/mL)**	6.7%	0.5%	8%
**Creatinine-D3 (5 µg/mL)**	3.7%	0.4%	12%
**Blood spheroid**			
**Spiked creatinine (0, 2.5–20 µg/mL)**	6.8%	0.7%	10%
**Spiked creatinine-D3 (5 µg/mL)**	3.9%	0.4%	11%

Supplemental Table S2 SRM response of internal standard measured in the blank hydrophobic paper and the dried horse whole blood spheroid sample with the matrix effect expressed in percentages.

Responses (mean peak area)	2.5 µg/mL	5 µg/mL	10 µg/mL	20 µg/mL
**Blank hydrophobic paper (CV)**	17469654 (15%)	25464335 (4%)	48229141 (6%)	51647044 (14%)
**Dried blood spheroid (CV)**	2509014 (35%)	2812771 (22%)	5747822 (34%)	6904991 (28%)
***Matrix effect**	-86%	-89%	-88%	-87%

*Matrix effect was expressed as the ratio of the mean peak area from the dried blood spheroid to the mean peak area from the blank hydrophobic paper multiplied by 100. A value of >100% indicates ionization enhancement, and a value of <100% indicates ionization suppression.

Supplemental Table S3 Comparison of creatinine concentrations in real human blood samples between different detection and calibration methods.

Detection method	Calibration method	Mean calculated creatinine concentration ± SD (µg/mL)		
		Individual 1	Individual 2	Individual 3
**PSI-MS/MS (whole blood)**	**External calibration**	8.62 ± 0.39	6.48 ± 0.37	7.61 ± 0.30
**PSI-MS/MS (whole blood)**	**Standard addition**	8.86 ± 0.31	6.31 ± 0.26	7.33 ± 0.30
**UPLC-MS/MS (whole blood)**	**Standard addition**	8.91	5.91	7.04
**UPLC-MS/MS (plasma)**	**Standard addition**	8.22	5.72	7.24

Supplemental Table S4 Comparison of estimated average cost, sample preparation requirement, analysis running time and environmental considerations for PSI-MS/MS, UPLC-MS/MS, Jaffé and enzymatic methods.

	**PSI-MS/MS ***	**UPLC-MS/MS**	**Jaffé assay #**	**Enzymatic assay ^**
**Estimated average cost**	< ~£0.1 per sample (silanized paper substrate)	> ~£1 per sample (HPLC inserts, caps and UPLC column)	> ~£7 (assay kits and 10kDa centrifugal filter)	> ~£19 (assay kits and 10kDa centrifugal filter)
**Treated sample requirement (per sample)**	~5 µL IS-spiked whole blood	~50 µL IS-spiked whole blood	15 µL deproteinated blood	100 µL deproteinated blood (50 /100 µL to measure endogenous interference: sarcosine and creatine)
**Sample preparation time**	~12 min (drying in the oven)	~80 min (deproteination using methanol and centrifugation)	~15 min (deproteination using filter and centrifugation)	~15 min (deproteination using filter and centrifugation)
**Other preparation time**	-	~60 min (preparation of mobile phase and UPLC-MS system pre-conditioning)	~60 min (equilibration of cooled reagents at room temperature, and mixing reagents before use)	~60 min (equilibration of frozen reagents at room temperature and mixing reagents before use)
**MS running time / incubation time for reaction**	~2 min	~5 min	~7 min	~ 60 min
**Environmental considerations**	Biodegradable substrate and minimal solvent usage (~60 µL per sample)	Plastic and glass consumables and periodic column change. High solvent usage: > ~1.5 mL per sample.	Plastic consumables, waste and storage treatment of picric acid which is explosive in a dry state. Solvent usage: ~200 µL per sample.	Plastic consumables. Solvent usage: ~100 µL per sample.

* The initial capital cost for a mass spectrometer can be quite significant. However, since mass spectrometers are ubiquitous to the clinical lab (being used for a range of other tests), we have not included this cost in our preliminary analysis.

# Based on creatinine (serum) colorimetric assay kit (number 700460, Cayman Chemical, Ann Arbor, MI).

^ Based on creatinine assay kit (Catalog Number MAK080, Sigma, St. Louis, MO).

Supplemental Figure S1 (1) Water contact angles and (2-4) SEM images of (a) normal chromatographic paper and (b) hydrophobic silanized paper magnified at (2) 500x, (3) 1500x, (4) 5000x. As can be seen in the SEM images, there is evidence of a reduction in the number of pores and fibres on the hydrophobic paper substrate (b2-4).

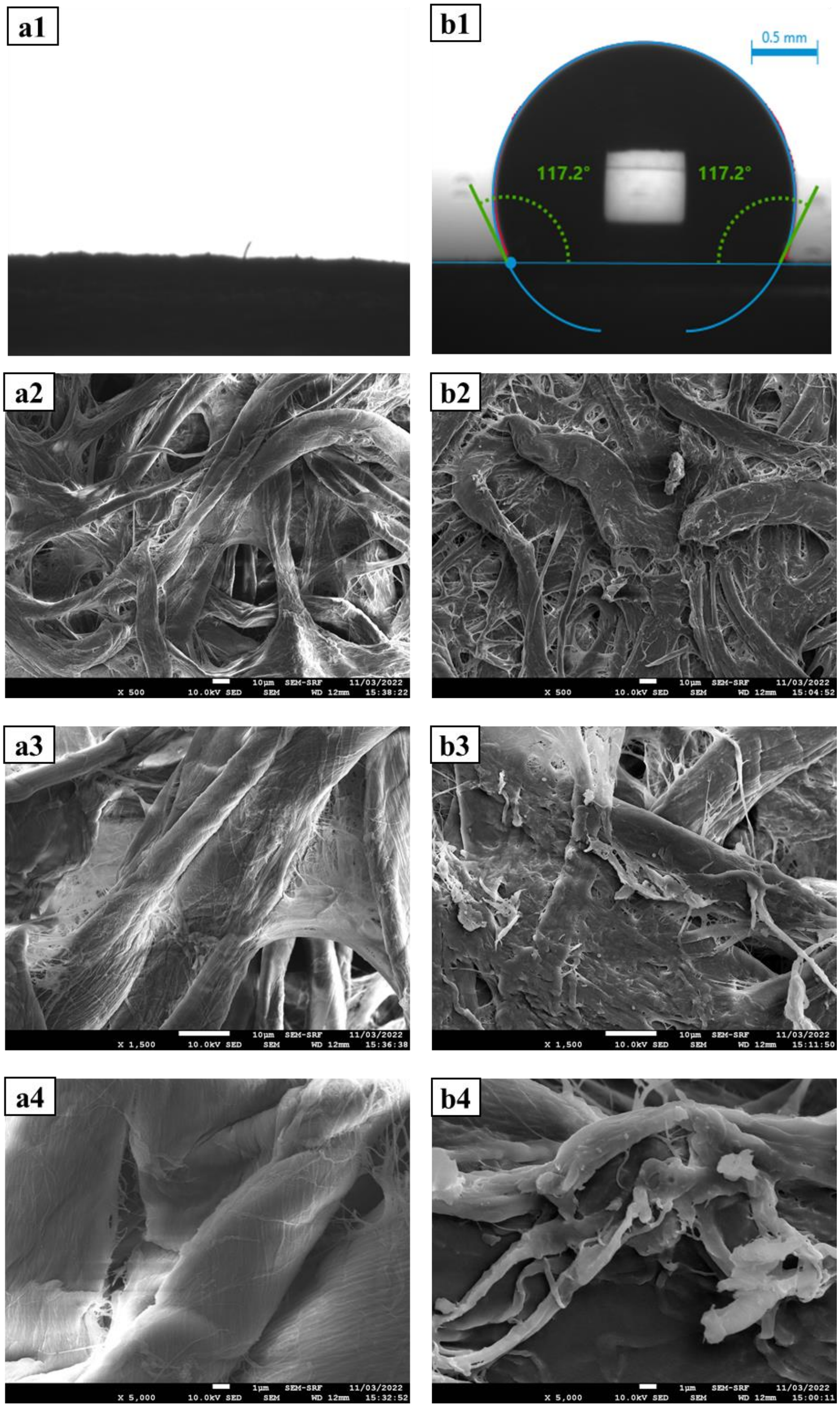

Supplemental Figure S2 Standard addition of peak area ratios of 0 and 2.5-20 µg/mL creatinine-D3 to unspiked, endogenous creatinine in horse whole blood.

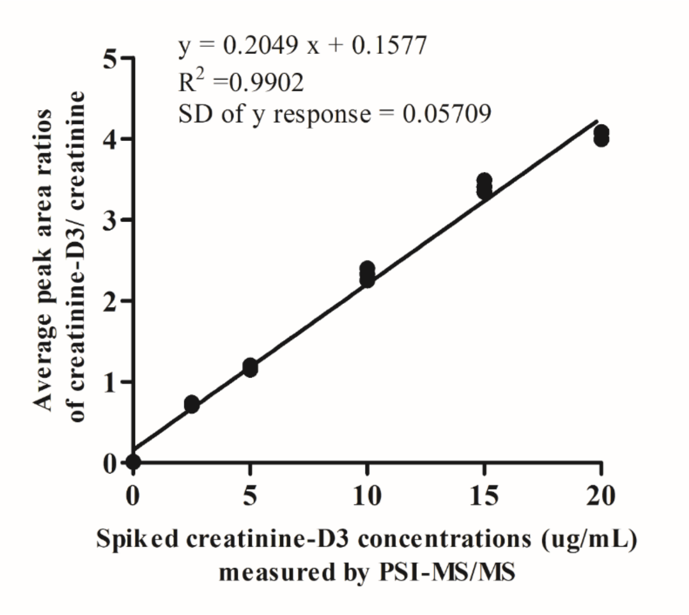

Supplemental Figure S3 Comparison of external calibration and standard addition of peak area ratios of creatinine to creatinine-D3 in horse whole blood spheroid samples by PSI-MS/MS.

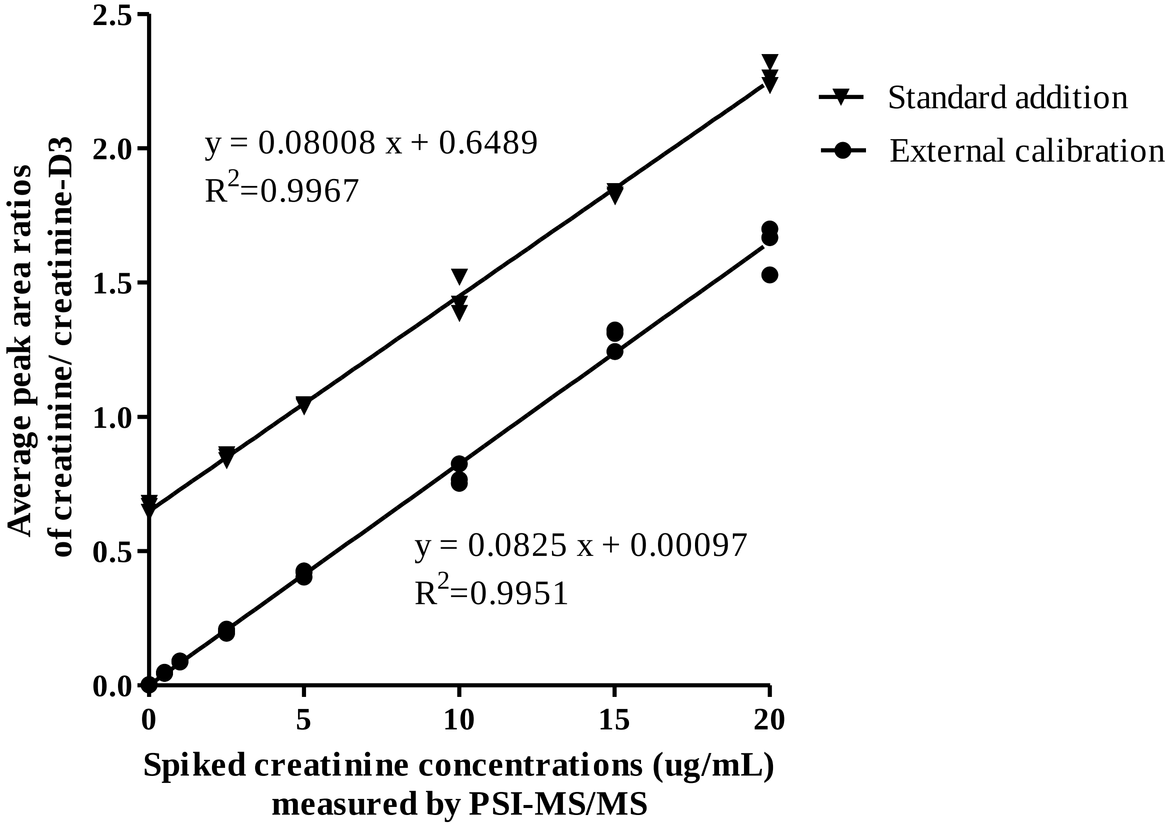

Supplemental Figure S4 Representative PSI-MS/MS SRM chromatograms of (1) quantifier and (2) qualifier ion transitions selected for (a) creatinine and (b) creatinine-D3 in a whole blood sample, dried on silanized paper from a healthy volunteer and spiked with 5 μg/mL creatinine-D3.

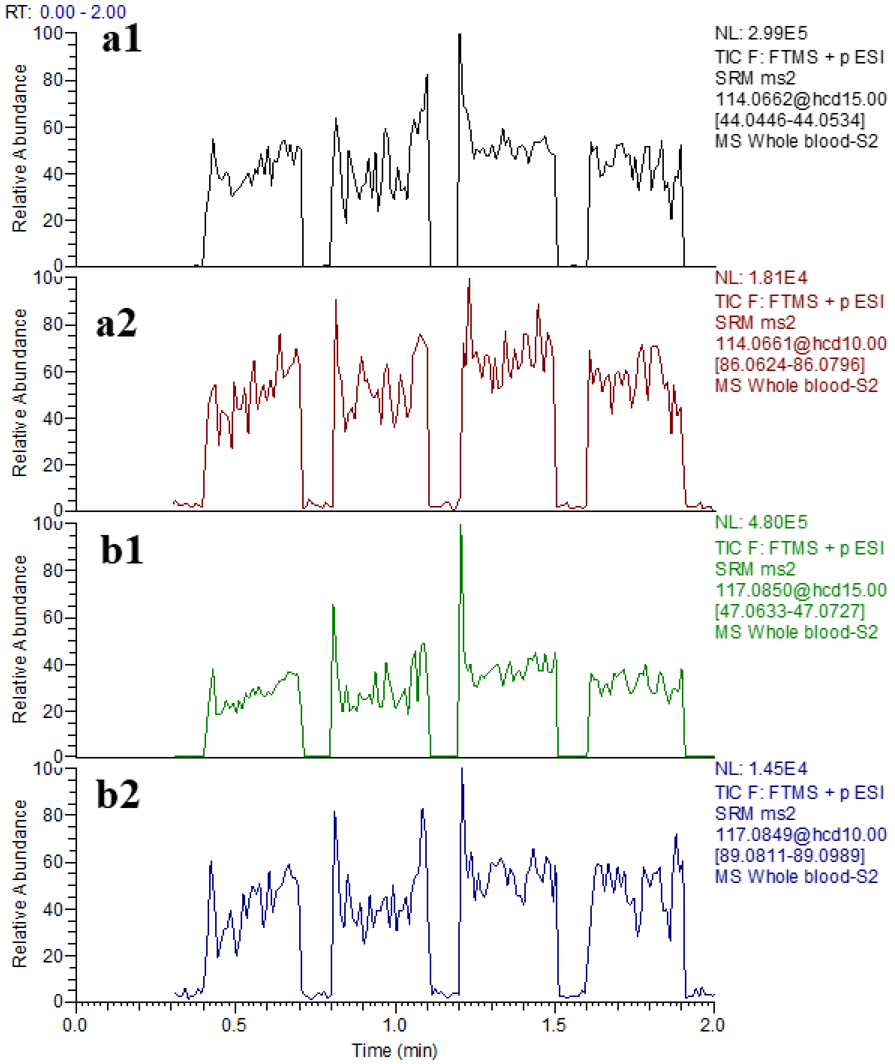

Supplemental Figure S5 (a) Representative PSI-MS/MS SRM chromatograms of the quantifier ion transition for creatinine and (b) average full scan mass spectra of (1) blank water, (2) a neat aqueous calibration solution (10 µg/mL creatinine and 5 µg/mL creatinine-D3), and (3) a human whole blood sample spiked with 5 µg/mL creatinine-D3.

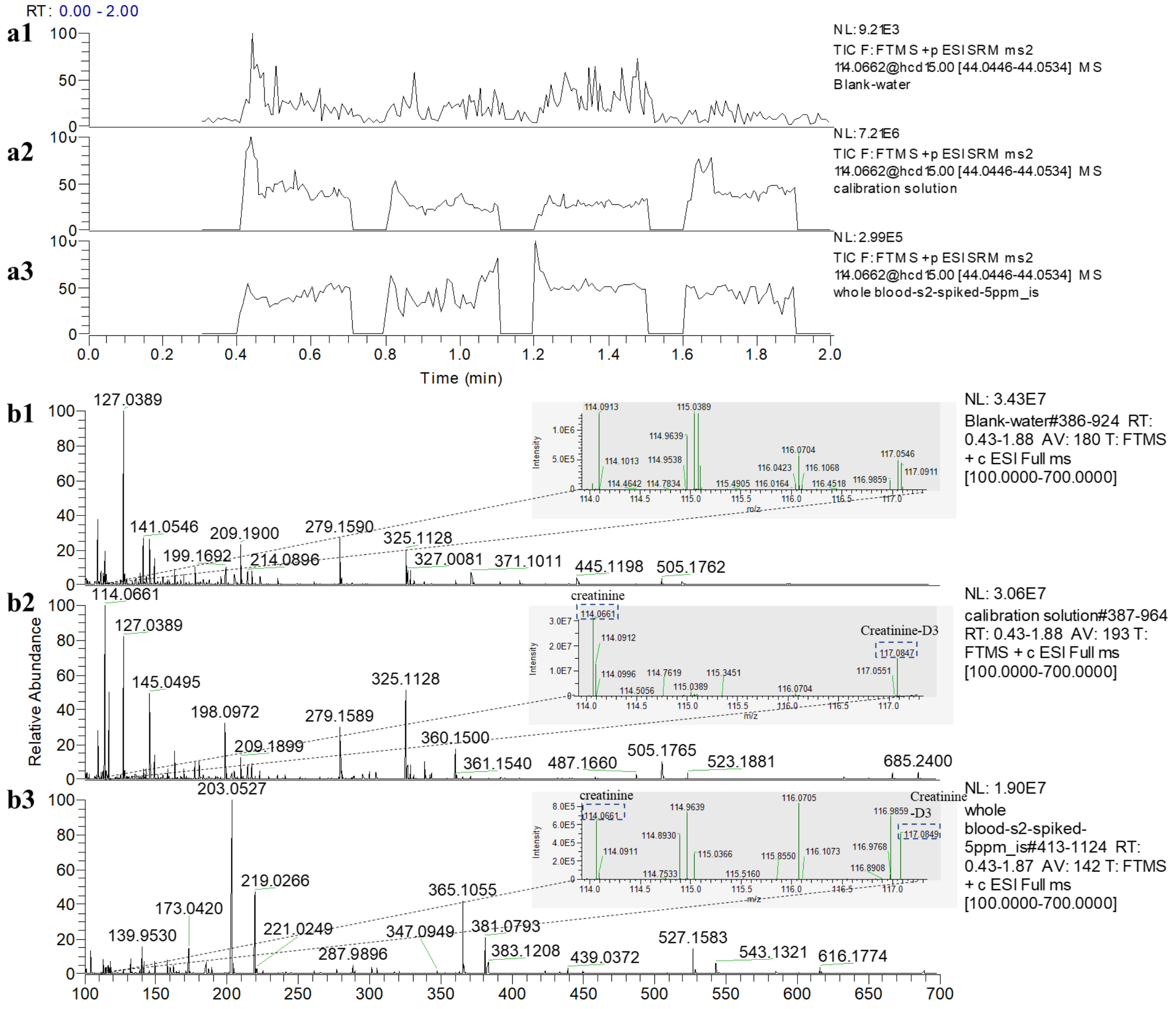

Supplemental Figure S6 Comparison of creatinine concentrations in real human blood samples between unsilanized and silanized paper. Data are expressed as mean ± SD. *p* value: Student's t-test (95% confidence interval).

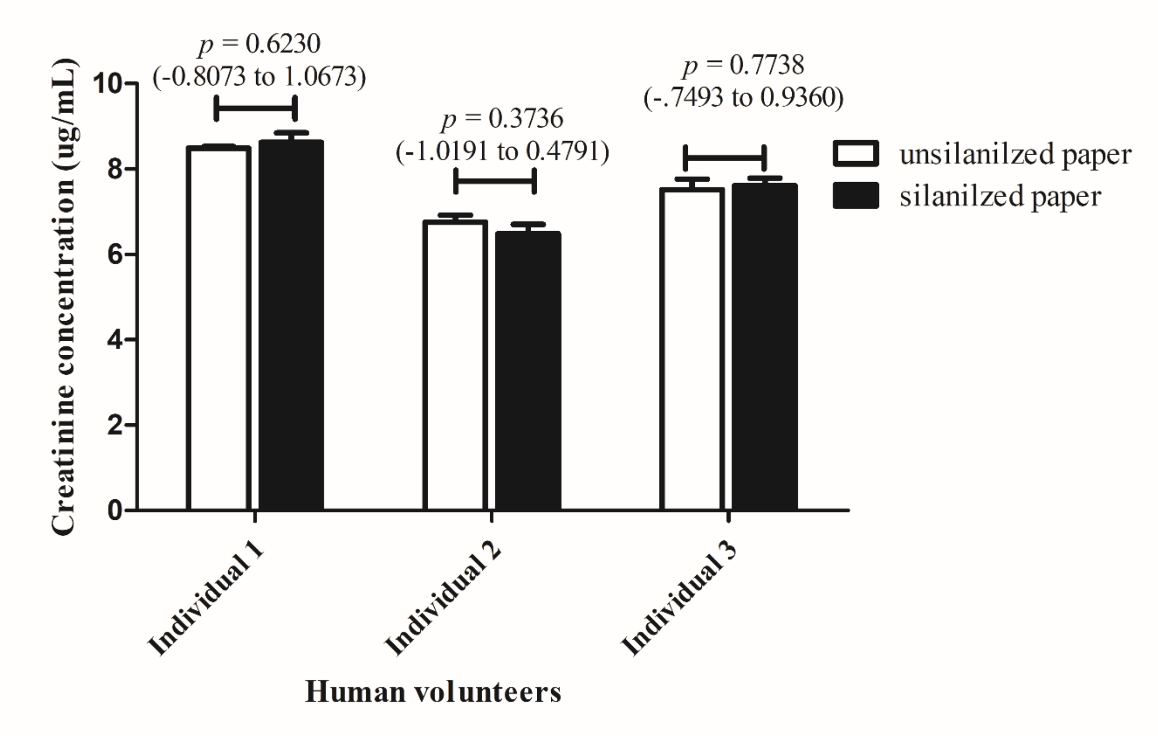

Supplemental Figure S7 Representative UPLC-MS/MS MRM chromatograms of (1) quantifier and (2) qualifier ion transitions selected for (a) unspiked creatinine and (b) 5 µg/mL of spiked creatinine-D3 measured in a human whole blood sample.

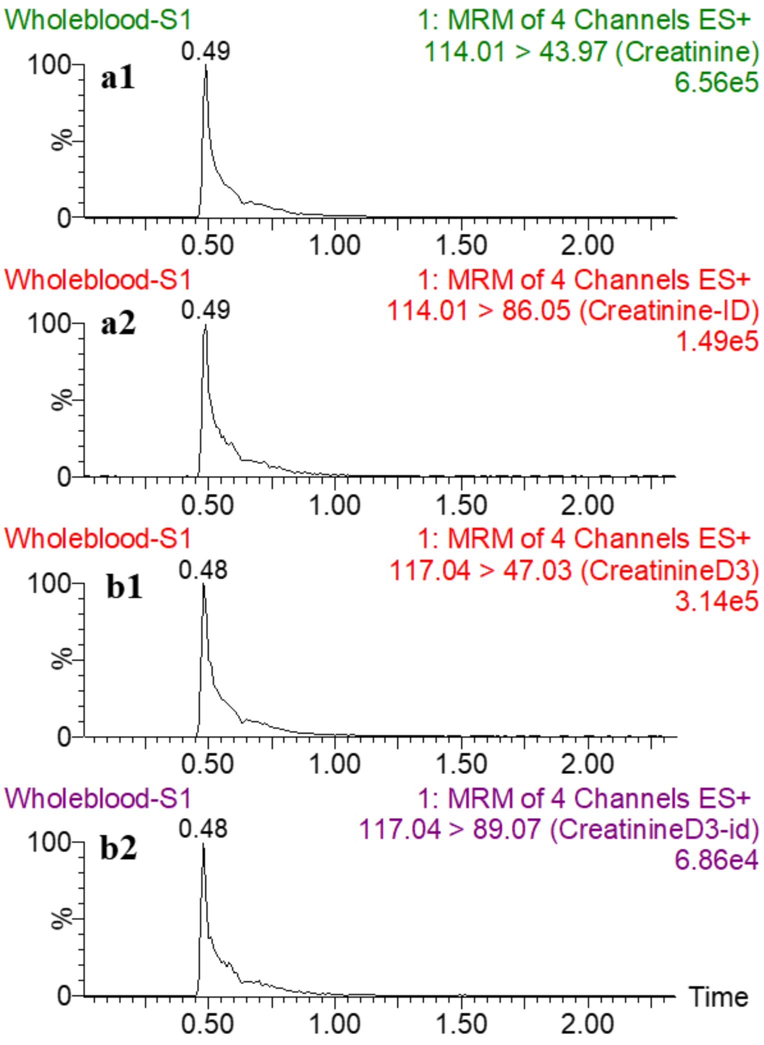

Supplemental Figure S8 Calibration curves of standard addition of (a) spiked human whole blood and (b) plasma samples of the three individuals (1, Individual 1; 2, Individual 2; 3, Individual 3) using UPLC-MS/MS.

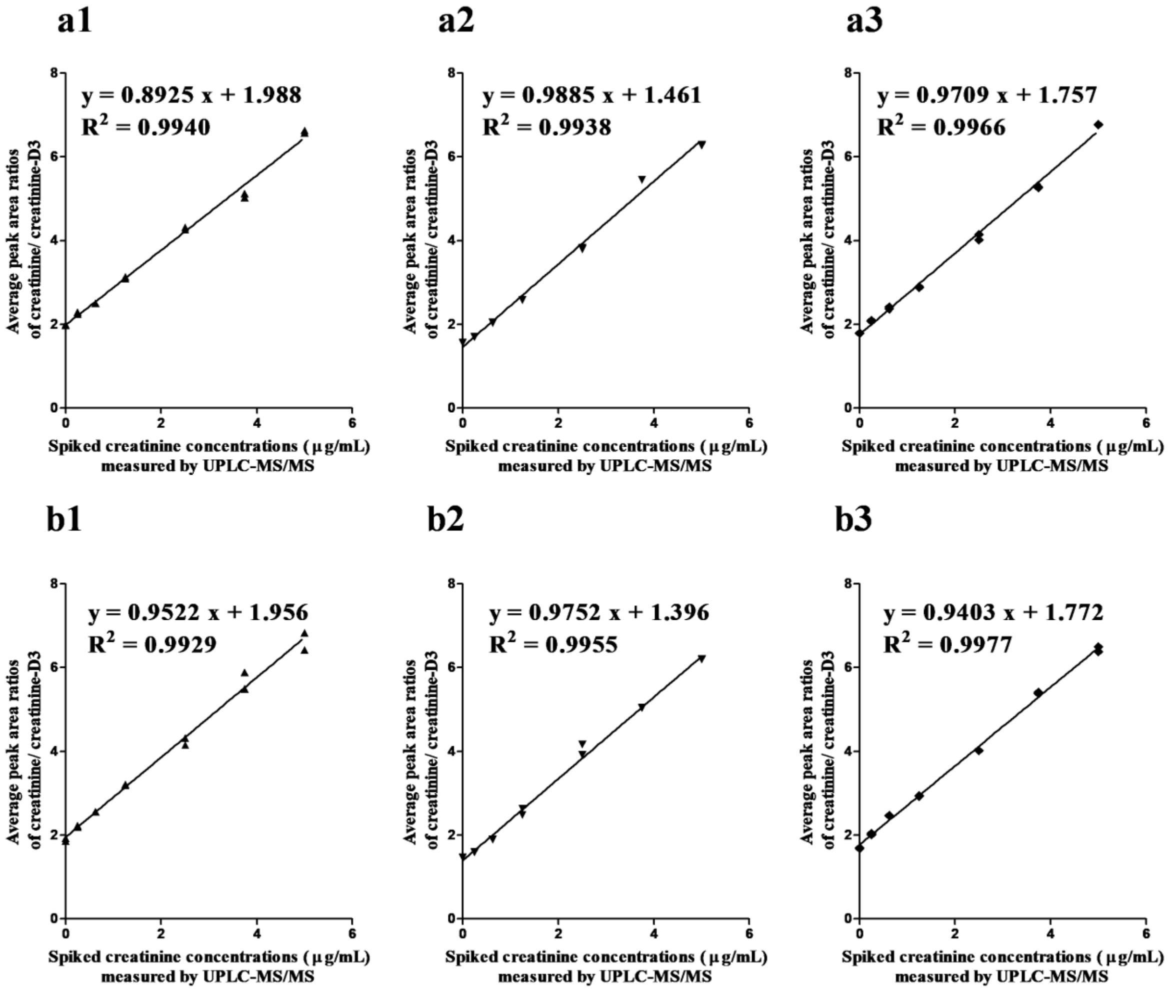

Supplement: Supplementary file 1 — Supplementary Information. [file 41598_2022_18365_MOESM1_ESM.pdf]
